# Amussat’s Laxative Syrup

**Published:** 1882-11

**Authors:** 


### Amussat's Laxative Syrup.

In the *Gazette Hebdomadaire*, 1882, No. 22 (*Lyon Medical*, June 4, 1882), we find the following formula for the preparation of the "sirop laxatif d'Amussat," or "sirop de suc d'herbes":

Rasped guaiacum wood,
Chicory root,
Burdock root,
Waterdock root (racine de patience),
Fumitory tops,
Tops of viola tricolor arvensis (pensée sauvage), each, 100 grammes,
Senna leaves, 500 grammes.

Bruise the materials, and infuse for twelve hours with five kilogrammes of boiling water. Strain, and make a second infusion with three kilogrammes of water. Strain under pressure, filter through paper, and make, with honey and sugar, each, three kilogrammes, a syrup, which is also to be filtered through paper, and which should be of the density of 31° Baumé. Dose, one to two tablespoonfuls a day.—*N. Y. Med. Jour. and Obst. Rev.*

